# Advances in astrocytic calcium signaling research

**DOI:** 10.3389/fncel.2025.1719532

**Published:** 2025-11-26

**Authors:** Yuzhu Chen, Yejun Ye, Joyce Jia, Binhao Long, Tingting Dou, Xingke Yan

**Affiliations:** 1 Department of Traditional Chinese Medicine, Qinghai University School of Medicine, Xining, Qinghai, China; 2 College of Acupuncture and Tuina, Gansu University of Chinese Medicine, Lanzhou, Gansu, China

**Keywords:** astrocyte calcium signaling, review, astrocyte, calcium, calcium signaling

## Abstract

Astrocytes are the most abundant glial cells in the central nervous system. They detect neuronal activity through Ca^2+^ signals and thereby regulate synaptic plasticity, integrate neuronal information, and maintain extracellular homeostasis. Growing evidence indicates that aberrant astrocytic Ca^2+^ signaling is an important pathological factor in the onset and progression of many neurological disorders. In this review, we systematically summarize the sources, classifications, detection methods, and functional significance of astrocyte Ca^2+^ signaling, with the aim of improving understanding of astrocyte function and providing new perspectives and rationale for therapeutic strategies targeting related diseases.

## 1 Introduction

Astrocytes are the most widely distributed, numerous, and largest type of glial cell in the central nervous system. By buffering extracellular ions, clearing neurotransmitters, and supplying metabolic substrates to neurons, they play a central role in maintaining brain homeostasis and information processing (Ahmadpour et al., 2021). In addition, astrocytes secrete a variety of signaling molecules—including neurotransmitters, precursor molecules, hormones, and growth factors—and participate in the regulation of circadian rhythms, sleep–wake cycles, learning and memory, and other related behaviors (Li X. et al., 2025; Nagai et al., 2021). Unlike neurons, astrocytes do not generate action potentials; instead, their intracellular Ca^2+^ concentration changes at rest or in response to external stimuli, referred to as Ca^2+^ signals. In recent years, driven by the development of genetically encoded calcium indicators and advanced imaging technologies, research on astrocytic Ca^2+^ signaling has progressed markedly (Semyanov et al., 2020). Studies show that astrocytic Ca^2+^ signals are not confined to the soma but are widespread in subcellular compartments such as processes and endfeet, exhibiting pronounced spatiotemporal heterogeneity (Lia et al., 2021). These Ca^2+^ signals participate in neurotransmitter release, modulation of neuronal excitability, local energy metabolism, and neurovascular coupling, and therefore play important roles in information integration and functional homeostasis of neuronal networks (Fernández-González and Galea, 2023; Herrera Moro Chao et al., 2022; Denizot et al., 2019). Accordingly, investigation of Ca^2+^ signaling may help inform therapeutic strategies for related disorders. This review summarizes the sources, classification, detection methods, and functional significance of astrocytic Ca^2+^ signaling, with the aim of providing a reference for elucidating astrocyte function and potential intervention approaches.

## 2 Sources and classification of astrocytic Ca^2+^ signals

### 2.1 Sources of Ca^2+^ signals

Astrocytic intracellular Ca^2+^ signals originate from diverse sources, broadly categorized into internal stores and extracellular influx. The major intracellular reservoirs include the endoplasmic reticulum, mitochondria, and lysosomes, which regulate cytosolic Ca^2+^ homeostasis and dynamics through release and reuptake mechanisms. Extracellular Ca^2+^ entry, on the other hand, is mediated by multiple membrane channels and receptors, such as transient receptor potential (TRP) channels, N-methyl-D-aspartate (NMDA) receptors, sodium–calcium exchangers (NCX), and voltage-gated Ca^2+^ channels (VGCCs). These pathways enable Ca^2+^ influx in response to membrane depolarization or neurotransmitter stimulation, thereby shaping the spatiotemporal profile of astrocytic Ca^2+^ signaling.

#### 2.1.1 Endoplasmic reticulum

The endoplasmic reticulum (ER) represents the principal intracellular Ca^2+^ store in astrocytes, where Ca^2+^ release is predominantly mediated by inositol 1,4,5-trisphosphate receptors (IP3Rs) and ryanodine receptors (RyRs), generating spatiotemporal Ca^2+^ dynamics that extend from the soma to fine processes (Giorgi et al., 2018; Vandecaetsbeek et al., 2011; Raffaello et al., 2016). Stobart et al. (2018a) demonstrated that genetic deletion of IP3R2 markedly reduced multimodal Ca^2+^ signals in layer II/III of the somatosensory cortex following sensory stimulation, underscoring the pivotal role of IP3R2 in astrocytic Ca^2+^ dynamics. In awake mice, Srinivasan (Srinivasan et al., 2015) reported that astrocytic Ca^2+^ signals in the visual cortex can be divided into rapid and delayed components; notably, IP3R2 deficiency abolished fast somatic signals without affecting delayed responses, suggesting distinct sources of Ca^2+^ between soma and processes. Consistent with this, subsequent studies confirmed that somatic Ca^2+^ dynamics are primarily dependent on ER-localized IP3R2, whereas local Ca^2+^ fluctuations within processes may be mediated by alternative channels or compartment-specific mechanisms (Cresswell-Clay et al., 2018). Nevertheless, high spatiotemporal resolution imaging has revealed IP3R2-independent microdomain events within processes, indicating substantive differences in the origin and amplification mechanisms of somatic versus process Ca^2+^ signals (Bindocci et al., 2017).

In addition to IP3Rs, ER release is amplified through Ca^2+^-induced Ca^2+^ release (CICR) mediated by RyRs, which also contribute to intercellular signal propagation among neighboring astrocytes (Weiss et al., 2022). Pharmacological evidence further supports this role: the RyR agonist caffeine enhances both spontaneous and evoked microdomain Ca^2+^ activity, whereas the RyR blocker ryanodine reduces such activity (Lalo and Pankratov, 2024). Moreover, ER contact sites with other organelles—particularly mitochondria-associated membranes (MAMs)—facilitate efficient ER-to-mitochondria Ca^2+^ transfer by shortening the inter-organelle distance and enriching IP3Rs together with associated channel complexes (Dematteis et al., 2024).

#### 2.1.2 Mitochondria

Mitochondria are abundant in astrocytes and precisely shape local Ca^2+^ dynamics by sequestering and releasing cytosolic Ca^2+^ while coupling these signals to cellular energy metabolism (Aten et al., 2022). Mitochondrial Ca^2+^ uptake is primarily mediated by the mitochondrial calcium uniporter (MCU) complex and its regulatory subunits (e.g., MICU1/2, EMRE), whereas Ca^2+^ extrusion depends largely on the mitochondrial Na^+^/Ca^2+^ exchanger (NCLX) and, under severe stress, transient opening of the mitochondrial permeability transition pore (mPTP). Together, these pathways determine the mitochondrion’s roles in shaping fast peak responses and longer-term Ca^2+^ homeostasis (Feno et al., 2021; Vercesi et al., 2018). Uptake of Ca^2+^ into mitochondria activates several dehydrogenases, thereby stimulating the tricarboxylic acid cycle and ATP production to support astrocyte–neuron metabolic coupling and synaptic plasticity; conversely, excessive mitochondrial Ca^2+^ promotes reactive oxygen species (ROS) generation and can trigger mPTP opening, leading to energetic failure and functional impairment (Modesti et al., 2021). Experimental manipulations that inhibit MCU or dissipate the mitochondrial membrane potential typically reduce mitochondrial Ca^2+^ clearance and are associated with larger cytosolic Ca^2+^ peaks, whereas NCLX inhibition impairs mitochondrial Ca^2+^ efflux and perturbs downstream metabolic pathways (Reyes and Parpura, 2008).

Importantly, conclusions about mitochondrial Ca^2+^ influx and efflux depend critically on the choice of mitochondrial Ca^2+^ indicators (e.g., mito-GCaMP variants, 4mtD3cpv, ROMO-GemGeCO), imaging resolution, and experimental context (*in vitro* slices versus *in vivo* preparations). Therefore, interpretations of somatic versus microdomain mitochondrial Ca^2+^ dynamics should always consider these methodological constraints.

#### 2.1.3 Lysosomes

Lysosomes constitute an important intracellular Ca^2+^ reservoir in astrocytes, with luminal Ca^2+^ concentrations reaching several hundred micromolar, a level that is highly dependent on luminal acidification. Proton gradients generated by vacuolar-type H^+^-ATPase (V-ATPase) drive H^+^/Ca^2+^ exchange and thereby enable lysosomal Ca^2+^ uptake (Churchill et al., 2002; Ronco et al., 2015). Nicotinic acid adenine dinucleotide phosphate (NAADP) functions as a potent “trigger” to evoke lysosomal Ca^2+^ release, which can then be amplified through recruitment of ER ryanodine receptors (RyRs) to produce broader intracellular Ca^2+^ waves in accordance with the trigger hypothesis (Galione, 2015; Penny et al., 2015). On the lysosomal membrane, the mucolipin channel TRPML1 mediates cationic efflux including Ca^2+^, and has been shown to modulate mitochondrial Ca^2+^ dynamics via lysosome–mitochondria signaling coupling (Li et al., 2017; Schmiege et al., 2017; Chen et al., 2017; Hirschi et al., 2017; Fine et al., 2018). For example, activation of TRPML1 with ML-SA1 produces sustained increases in mitochondrial Ca^2+^—including higher peak and mean mitochondrial Ca^2+^ levels across multiple time points—consistent with TRPML1-driven lysosomal Ca^2+^ export functionally engaging mitochondria (Peng et al., 2020).

Two-pore channel 2 (TPC2) is another lysosomal channel activated by NAADP and phosphatidylinositol 3,5-bisphosphate [PI(3,5)P2]; its ion selectivity is agonist-dependent, permitting either Ca^2+^ or Na^+^ permeability under different conditions (Zong et al., 2009; Cang et al., 2013; Gerndt et al., 2020). Imaging studies indicate that low concentrations of a TPC2 agonist (e.g., 10 μM A1N) produce modest cytosolic Ca^2+^ increases without depleting thapsigargin (TG)-sensitive ER stores, whereas higher concentrations (e.g., 30 μM A1N) can elicit robust cytosolic Ca^2+^ elevations and render cells unresponsive to subsequent TG, implying dose-dependent effects on lysosomal permeability and lysosome–ER Ca^2+^ coupling (Wang Q. et al., 2023). Overall, the strength and temporal profile of lysosome–ER–mitochondria Ca^2+^ coupling are highly sensitive to agonist dose, lysosomal luminal pH, and the depletion state of ER Ca^2+^ stores; therefore, experimental conditions must be carefully controlled and reported when interpreting dose-dependent or threshold phenomena in organelle Ca^2+^ signaling.

#### 2.1.4 Transient receptor potential (TRP) channels

Transient receptor potential (TRP) channels are non-selective cation channels with notable Ca^2+^ permeability and play important roles in both physiological and pathological astrocyte functions (Lia et al., 2021; Ahmadpour et al., 2021). TRP channels are activated by diverse stimuli — including mechanical stretch, osmotic changes, temperature, and lipid or chemical ligands — and multiple TRP subtypes are expressed in astrocytes (e.g., TRPA1, TRPC1/4–6, TRPV1, TRPV4) (Verkhratsky et al., 2014; Verkhratsky and Nedergaard, 2018). These subtypes contribute selectively to local Ca^2+^ homeostasis and synaptic regulation: for example, TRPA1 participates in hippocampal Ca^2+^ activity regulation (Shigetomi et al., 2013; Shigetomi et al., 2011), TRPC6 influences neuronal survival and synaptogenesis (Quick et al., 2012), and TRPV4 mediates responses related to local blood flow and external stimulation (Sucha et al., 2022; Tureckova et al., 2023). Functional perturbation studies further support subtype-specific roles. Shigetomi (Shigetomi et al., 2013) showed that the TRPA1 blocker HC-030031 reduces astrocytic Ca^2+^ signals, and genetic targeting of Trpa1 lowers the frequency of spontaneous astrocytic Ca^2+^ events, implicating TRPA1 in basal Ca^2+^ influx. Cheng (Cheng et al., 2023) used *in vivo* Ca^2+^ imaging to demonstrate that HC-030031 markedly decreases spontaneous hippocampal astrocyte Ca^2+^ events, with an even greater suppressive effect following social isolation — suggesting that stressors can upregulate TRPA1 function and thereby enhance astrocytic Ca^2+^ activity. Similarly, in models of intracerebral hemorrhage, reactive astrocyte proliferation and elevated Ca^2+^ activity are attenuated by Trpa1 knockout, highlighting TRPA1 as a key regulator of injury-related astrocytic Ca^2+^ influx (Xia et al., 2023). TRPV4 exhibits a distinct functional profile: the TRPV4 agonist GSK1016790A increases spontaneous Ca^2+^ activity in astrocyte endfeet, whereas the TRPV4 antagonist HC-067047 does not significantly alter basal spontaneous events but effectively suppresses endfoot Ca^2+^ responses to external stimulation (Dunn et al., 2013). These observations indicate that TRPV4 is preferentially engaged by exogenous stimuli, while spontaneous microdomain activity is more reliant on endogenous Ca^2+^ release mechanisms. Collectively, these findings underscore the heterogeneity of TRP channel contributions to astrocytic Ca^2+^ signaling and emphasize the need to consider both subtype and context (basal versus evoked; physiological versus pathological) when interpreting their roles.

#### 2.1.5 N-methyl-D-aspartate (NMDA) receptors

N-methyl-D-aspartate (NMDA) receptors, prototypical ionotropic glutamate receptors, are increasingly recognized to be functionally expressed on astrocyte membranes and to mediate Ca^2+^ influx into astrocytes (Ahmadpour et al., 2021). Multiple studies have provided direct evidence for the functional expression of NMDARs in astrocytes. Astrocytes express functional NMDARs that can evoke inward currents and Ca^2+^ signals (Lalo et al., 2006; Latour et al., 2001), and the Ca^2+^ signaling of CA1 astrocytes exhibits regional and subtype-specific functional heterogeneity (Serrano et al., 2008). Mechanistically, activation of astrocytic NMDARs induces a slow depolarization, which in turn opens L-type voltage-gated Ca^2+^ channels (L-VGCCs) and amplifies Ca^2+^ signaling. Blocking GluN1, inhibiting L-VGCCs, or preventing astrocytic depolarization eliminates the baseline heterogeneity of paired-pulse ratio (PPR) across convergent inputs (Letellier et al., 2016). Further evidence has shown that astrocytic NMDARs containing the GluN2C subunit maintain a broad distribution of presynaptic release probabilities, thereby preserving the coexistence of strong and weak synapses and facilitating both LTP and LTD without altering the mean synaptic strength (Chipman et al., 2021). In line with these findings, physiological stimulation paradigms (for example, theta-burst firing or focal glutamate application) can produce brief Ca^2+^ transients in subsets of astrocytes followed by a shifted, lower steady-state cytosolic Ca^2+^; these effects are antagonized by NMDAR blockers such as dl-APV. Intracellular loading of MK-801 into astrocytes abolishes theta-burst-induced Ca^2+^ decreases, whereas extracellular application alone is insufficient, indicating that blockade of astrocytic (rather than exclusively neuronal) NMDARs is necessary to eliminate this response and supporting a cell-autonomous role for astrocytic NMDARs in shaping activity-dependent Ca^2+^ dynamics (Mehina et al., 2017). Moreover, Na^+^ influx in hippocampal and cortical circuits can drive the sodium–calcium exchanger (NCX) into reverse mode, thereby amplifying intracellular Ca^2+^ elevation and producing a compound ionic response downstream of NMDAR activation (Ziemens et al., 2019; Benz et al., 2004). Importantly, the contribution of astrocytic NMDARs is highly heterogeneous: it varies across brain regions, developmental stages, and experimental contexts (*in vivo* versus *in vitro*). For example, blockade with D-AP5 did not alter spontaneous microdomain Ca^2+^ events in hippocampal astrocytes in some imaging studies, suggesting that astrocytic NMDARs may be selectively engaged during nearby synaptic activity rather than during basal microdomain signaling (Haustein et al., 2014). Complementary loss-of-function experiments (e.g., cortical Grin1 knockdown combined with membrane-tethered GCaMP imaging) show that reducing astrocytic NMDAR expression does not affect baseline spontaneous events or endfoot responses, but substantially impairs the recruitment, amplitude, and re-activation probability of soma and process Ca^2+^ microdomains evoked by stimulation—supporting the view that astrocytic surface NMDARs are critical for translating synaptic/afferent activity into localized somatic and process Ca^2+^ signals, whereas endfoot signaling relies more on alternative pathways (Ahmadpour et al., 2024). Taken together, these findings—and the ongoing debate over their physiological relevance—have been comprehensively summarized in recent reviews (Skowrońska et al., 2019; Verkhratsky and Chvátal, 2020).

#### 2.1.6 Sodium–calcium exchangers (Na^+^/Ca^2+^ exchangers, NCX)

The Na^+^/Ca^2+^ exchanger (NCX) is a pivotal membrane transporter in astrocytes that couples transmembrane electrochemical gradients via the exchange of three Na^+^ ions for one Ca^2+^ ion, thereby exerting powerful control over ionic homeostasis and cytosolic Ca^2+^ dynamics (Brandalise et al., 2023). NCX operates bidirectionally: in its forward mode (FNCX) it extrudes Ca^2+^ to help maintain low resting cytosolic Ca^2+^, whereas under conditions of elevated intracellular Na^+^ or altered membrane potential the exchanger can reverse (RNCX) and drive Ca^2+^ influx, contributing to cytosolic Ca^2+^ signaling. Patch-clamp studies have directly observed RNCX-associated currents concomitant with rises in intracellular Ca^2+^, whereas FNCX currents are less prominent under the same test conditions, indicating that forward-mode contribution to Ca^2+^ fluxes is contingent on specific Na^+^ gradients and membrane potentials (Rose et al., 2020; Wang et al., 2025). Mammalian NCX function is subject to Ca^2+^- and Na^+^-dependent allosteric regulation that is primarily mediated by two intracellular Ca^2+^-binding domains, CBD1 and CBD2 (Ottolia et al., 2021; Hilgemann, 2020). CBD1 contains multiple high-affinity Ca^2+^ binding sites (Kd ≈ 0.3 μM) and acts as a rapid Ca^2+^ sensor to activate exchanger current, while CBD2’s affinity and stoichiometry are modulated by alternative splicing and can mitigate Na^+^-induced inactivation, thereby tuning exchanger responsiveness to local ionic environments (Khananshvili, 2023). Multiple mechanisms can elevate intracellular Na^+^ in astrocytes and thereby promote RNCX, including activation of ionotropic glutamate receptors (AMPA/NMDA), electrogenic uptake via excitatory amino acid transporters (EAATs), and GABA uptake by GAT-3. RNCX-mediated Ca^2+^ entry can amplify local Ca^2+^ signals and trigger gliotransmitter release (e.g., glutamate, ATP/adenosine, homocysteine), with consequent modulation of presynaptic neuronal activity (Ahmadpour et al., 2021). Nevertheless, the physiological significance of R_NCX as a primary source of astrocytic Ca^2+^ remains debated. There is clearer consensus that under pathological states characterized by massive Na^+^ accumulation—such as ischemia, reperfusion, or oxygen–glucose deprivation—NCX reversal contributes substantially to pathological cytosolic Ca^2+^ overload (Lenart et al., 2004; Annunziato et al., 2013; Gerkau et al., 2018).

#### 2.1.7 Voltage-gated Ca^2+^ channels (VGCCs)

**FIGURE 1:**
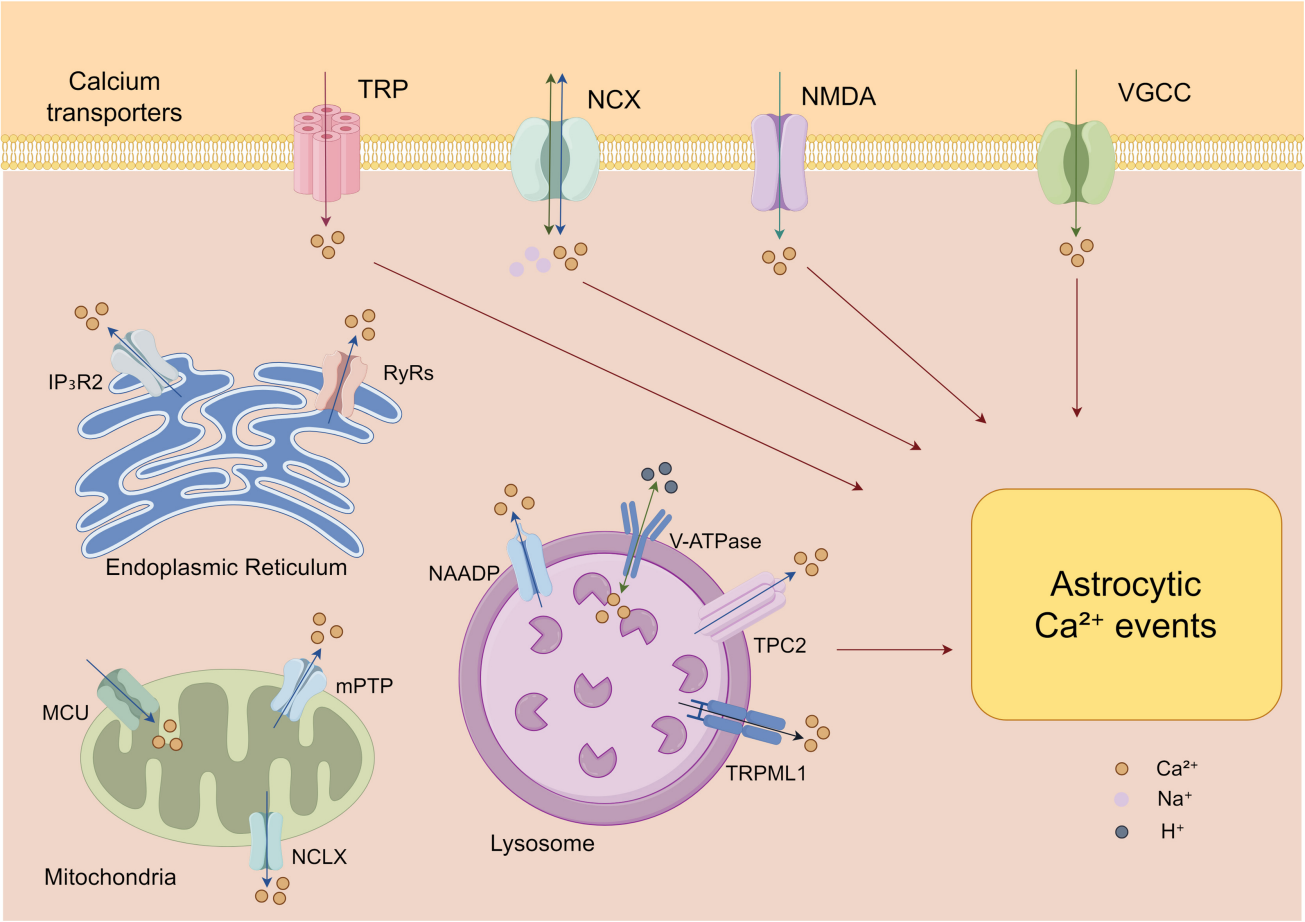
Astrocytes express diverse Ca^2+^ channels across subcellular compartments to regulate intracellular Ca^2+^ dynamics. Plasma membrane channels include TRP, NCX, NMDA receptors, and VGCCs, mediating Ca^2+^ influx. Intracellular stores such as the ER and mitochondria utilize IP3R2, RyRs, MCU, mPTP, and NCLX to control Ca^2+^ release and buffering. Lysosomal channels, including TPC2 and TRPML1, together with V-ATPase, maintain lysosomal Ca^2+^ homeostasis. This coordinated network supports astrocytic functions in synaptic modulation, energy metabolism, neuroinflammation, and neurovascular coupling. TRP, transient receptor potential; NCX, sodium-calcium exchanger; NMDA, N-methyl-D-aspartate; VGCC, voltage-gated Ca^2+^ channels; IP3R2, inositol 1,4,5-trisphosphate receptor type 2; RyRs, ryanodine receptors; MCU, mitochondrial calcium uniporter; mPTP, mitochondrial permeability transition pore; NCLX, mitochondrial sodium-calcium-lithium exchanger; NAAPD, Nicotinic acid adenine dinucleotide phosphate; V-ATPse, vacuolar-type H^+^-ATPase; TPC2, two-pore channel 2; TRPML1, transient receptor potential mucolipin 1; ER, endoplasmic reticulum (By Figdraw).

Astrocytes express voltage-gated Ca^2+^ channels (VGCCs). Although their overall expression is lower than in neurons, VGCCs can mediate Ca^2+^ entry during membrane depolarization and thereby constitute an important source of cytosolic Ca^2+^ dynamics in astrocytes (Denaroso et al., 2023). Multiple studies have systematically demonstrated that astrocytes possess functional voltage-gated calcium channels (VGCCs) under basal conditions. In primary cultured astrocytes, RT-PCR, Western blot, and immunocytochemistry analyses revealed the expression of multiple VGCC subtypes, including N-, L-, R-, and T-types, while P/Q-type channels were absent, providing molecular evidence for basal Ca^2+^ signaling (Latour et al., 2003). Patch-clamp recordings further confirmed that these channels generate functional currents even in the absence of external stimulation, indicating that VGCCs on the astrocyte membrane are active under basal conditions (D’Ascenzo et al., 2004). In acute hippocampal slices, L-type VGCCs were found to be expressed under basal conditions and could be activated by depolarization, leading to Ca^2+^ influx and regulating basal presynaptic strength heterogeneity of convergent synaptic inputs, highlighting the important role of basal VGCC activity in astrocytic Ca^2+^ signaling and synaptic function (Letellier et al., 2016). Specifically, the L-type channel Cav1.2 is a principal VGCC subtype implicated in depolarization-evoked Ca^2+^ influx (Cheli et al., 2016). Conditional deletion of Cav1.2 markedly attenuates high-K^+^-evoked astrocytic Ca^2+^ responses (by ∼28%), and pharmacological blockade with nitrendipine similarly abolishes high-K^+^-induced Ca^2+^ signals, implicating Cav1.2 as a core mediator of soma-level, depolarization-driven Ca^2+^ influx (Zamora et al., 2020). However, VGCC contributions are spatially heterogeneous: pharmacological studies indicate that VGCCs do not substantially drive spontaneous Ca^2+^ activity within fine astrocytic processes, suggesting that somatic Ca^2+^ transients rely more on Cav1.2 while process-localized microdomain activity depends predominantly on other Ca^2+^ sources (Rungta et al., 2016). Using the membrane Ca^2+^ indicator Fluo-4AM, Milorad Dragić and colleagues investigated the effects of trimethyltin (TMT) on Ca^2+^ dynamics in primary cultured cortical astrocytes. They found that TMT rapidly increased astrocytic Ca^2+^ concentration via L-type VGCCs and upregulated Cav1.2 protein expression, indicating that VGCC function and expression are enhanced under inflammatory conditions (Dragić et al., 2021). These findings suggest that astrocytic VGCCs may serve as potential therapeutic targets for mitigating inflammation and promoting brain recovery. Although multiple studies have demonstrated that astrocytes express VGCCs and contribute to cytosolic Ca^2+^ signaling, their expression levels, channel subtypes, and contributions to basal Ca^2+^ dynamics across different brain regions, developmental stages, and pathological states remain contentious and warrant further investigation (Lia et al., 2023). Figure 1 shows that astrocytes contain different Ca^2+^ channels.

### 2.2 Classification of Ca^2+^ signals

Distinct astrocytic subcellular compartments — soma (AS), processes (AP), distal fine processes (Gp), and perivascular endfeet (AE) — exhibit characteristic Ca^2+^ signaling profiles. Microdomains within fine processes produce fast, spatially restricted, second-scale events, whereas somatic signals manifest as lower-frequency, long-lasting, whole-cell responses. These compartment-specific spatiotemporal signatures likely reflect differences in local Ca^2+^ sources, amplification mechanisms, and functional roles (Institoris et al., 2022; Biesecker and Srienc, 2015).

#### 2.2.1 Microdomain Ca^2+^ signals

Microdomain Ca^2+^ signals in astrocytes denote spatially restricted, small-scale Ca^2+^ events that occur within fine processes or endfeet (Lia et al., 2021). Such microdomain activity is observed in both anesthetized and awake animals and persists even when neuronal activity and most neurotransmitter receptors are pharmacologically blocked, indicating that microdomain events can arise spontaneously (Takata and Hirase, 2008; Nimmerjahn et al., 2009). The origins of microdomain Ca^2+^ signals are heterogeneous, encompassing IP3R2-dependent ER release, mitochondrial Ca^2+^ efflux, and Ca^2+^ entry through membrane channels such as TRPV4 (Bai et al., 2024). Genetic and pharmacological work in invertebrate and vertebrate systems further illustrates this diversity: for example, a class of microdomain transients in Drosophila astrocytes is mediated by the TRP channel TrpML, is sensitive to reactive oxygen species and tyramine (via TyrRII), and requires extracellular Ca^2+^ while being TTX-insensitive—consistent with microdomains arising from both membrane channels and organelle–membrane coupling (Ma and Freeman, 2020; Bindocci et al., 2017). High-resolution 3D imaging studies localize the bulk of basal microdomain activity to fine processes, with relatively sparse somatic events; endfeet show Ca^2+^ signals but are often less frequent and less synchronized than processes (Bindocci et al., 2017). Microdomain events typically occur at higher frequency than somatic events but exhibit variable amplitudes and durations depending on brain region, indicator choice, and analysis methods. For instance, spontaneous process Ca^2+^ events in hippocampal cortex have reported half-widths (T0.5) on the order of ∼0.5–3.9 s and peak amplitudes reported in some studies as on the order of dF/F0; in hippocampal CA3 stratum lucidum, process events exhibited amplitudes ∼0.4 dF/F and durations of ∼6 s—metrics that can differ between studies owing to methodological factors (Shigetomi et al., 2010; Haustein et al., 2014). Critically, microdomain Ca^2+^ dynamics are tightly coupled to behavioral state and are implicated in the regulation of arousal, sleep, and locomotion (Wang F. et al., 2023). For example, astrocytic Ca^2+^ activity is markedly reduced during rapid eye movement (REM) sleep and increases upon awakening, underscoring a state-dependent modulation of microdomain signaling (Tsunematsu et al., 2021).

#### 2.2.2 Somatic Ca^2+^ signals

Beyond the fast, spatially restricted microdomain events in peripheral processes, astrocytes exhibit prominent somatic Ca^2+^ transients. Somatic events are typically low-frequency (∼0.01–0.03 Hz), high-amplitude (ΔF/F0 often ≥ 0.5) and long-lasting (T_0.5_ ≈ 5–15 s), and are largely driven by IP3R-mediated ER Ca^2+^ release. These whole-cell transients are markedly reduced or abolished in Itpr2^–/–^ (IP3R2 knockout) mice, indicating a strong dependency on IP3 signaling pathways (Fernández de la Puebla et al., 2025). Compared with microdomain activity, somatic dynamics are slower and may reflect spatiotemporal integration of multiple local microdomain events. Somatic Ca^2+^ signaling is tightly coupled to behavioral state and neuromodulatory tone. In awake animals, somatic Ca^2+^ increases are enhanced at movement onset and positively correlate with arousal metrics such as pupil diameter and locomotor speed (Reitman et al., 2023). Noradrenergic drive from the locus coeruleus (LC) is a potent trigger of broad astrocytic somatic Ca^2+^ responses: depletion of cortical norepinephrine with DSP4 or disruption of LC activity reduces startle-evoked astrocytic Ca^2+^ transients, implicating norepinephrine as a key neuromodulatory mediator (Ding et al., 2013). Strong sensory stimuli (e.g., startling inputs) reliably evoke somatic Ca^2+^ responses in wild-type animals but produce attenuated or absent responses in IP3R2-deficient mice, further supporting the central role of IP3R2-dependent ER release in behaviorally evoked whole-cell signals (Srinivasan et al., 2015).

Although microdomain and somatic Ca^2+^ signals differ markedly in their spatiotemporal profiles and triggering mechanisms, they are likely linked through hierarchical spatiotemporal integration to enable regulation across scales—from synaptic microcircuits to global brain state. Dissecting the causal relationships and functional consequences of microdomain–somatic conversions will require combined high spatiotemporal-resolution imaging and targeted perturbations to resolve how local events scale to whole-cell and network-level signaling. Table 1 presents a comparison of Ca^2+^ signals.

**TABLE 1:** Comparison of Ca^2+^ signals.

Comparison dimension	Microdomain Ca^2+^ signals	Somatic Ca^2+^ signals
Typical location	Fine processes, peripheral processes, endfeet	Soma (cell body)
Spatial scale (FWHM)	2–5 μm	Whole soma
Amplitude (ΔF/F_0_)	Low–medium (≈0.4–0.5)	High (≥0.5)
Duration (T_0.5_)	Short (0.5–3.9 s)	Long (5–15 s)
Primary triggers / mechanisms	Local channels (TRP, P2X, SOCE), mitochondria–ER coupling, local receptor activation	ER IP_3_R-mediated ER release, neuromodulators (e.g., norepinephrine) and other widespread activations
Relationship to behavior / state	Occur spontaneously; modulated by arousal, locomotion and sensory stimuli (↑ with movement/arousal)	Strongly dependent on behavioral state and arousal level (↑ at movement/onset)

## 3 Astrocytic Ca^2+^ signal detection and analysis

### 3.1 Ca^2+^ signal detection

Monitoring astrocytic Ca^2+^ signals requires careful consideration of both the biological indicators and the optical imaging platforms. The choice of calcium indicator determines the sensitivity, kinetics, and subcellular targeting of the detected signals, whereas the imaging system dictates the achievable spatial and temporal resolution as well as imaging depth.

#### 3.1.1 Calcium indicators

Calcium imaging using chemical or genetically encoded indicators provides a powerful approach to monitor intracellular Ca^2+^ dynamics in live cells.

##### 3.1.1.1 Chemical calcium indicators

Chemical calcium indicators are based on the BAPTA scaffold, which undergoes fluorescence intensity or spectral changes upon binding Ca^2+^, enabling real-time monitoring of intracellular Ca^2+^ dynamics (Csomos et al., 2021). According to their fluorescence properties and detection modalities, representative indicators can be classified into single-wavelength indicators (e.g., Fluo-4, OGB-1, Rhod-2) and ratiometric indicators (e.g., Fura-2, Indo-1) (Paredes et al., 2008; Bootman et al., 2013). Single-wavelength indicators exhibit strong fluorescence changes and high signal-to-noise ratios, but their readouts are often relative, sensitive to dye loading and photobleaching, making them suitable for monitoring microdomain Ca^2+^ signals in astrocytes, particularly in experiments requiring high spatial and temporal resolution (Bootman et al., 2013). In contrast, ratiometric indicators correct for experimental variability via excitation or emission wavelength ratios, enabling quantitative measurements of cytosolic Ca^2+^ concentrations (Bootman et al., 2013). For instance, Milićević (Milićević et al., 2022) used Fluo-4 to monitor astrocytic Ca^2+^ changes. Fluo-4 enters cells through the plasma membrane, where intracellular esterases cleave it to release the active dye, which distributes uniformly across soma and fine processes. The fluorescence changes reflect local Ca^2+^ variations, allowing monitoring of microdomain activity in processes and endfeet without relying on ratiometric measurements. Fluo-4 also enabled real-time observation of Ca^2+^ release and reuptake in ALS model rats, demonstrating its utility in detecting dynamic microdomain signals and pathological Ca^2+^ dysregulation. Conversely, Rhod-2 effectively labels astrocyte somata in the cortex but poorly visualizes fine processes and endfeet (Zhang et al., 2021; Thrane et al., 2012). Ratiometric indicators such as Fura-2, when excited at 340/380 nm and calibrated using Triton X-100/Ca^2+^ and EGTA, allow nM-level quantification of cytosolic Ca^2+^. This approach revealed concentration-dependent increases in astrocytic Ca^2+^ upon haloperidol treatment (Hsu and Liang, 2020). While Fura-2 provides robust quantitative measurements and mechanistic separation, it lacks subcellular spatial resolution. Therefore, combining ratiometric probes like Fura-2 with high spatiotemporal resolution single-wavelength indicators or GECI-based two-photon imaging is recommended to capture both quantitative precision and microdomain dynamics.

##### 3.1.1.2 Genetically encoded calcium indicators (GECIs)

Genetically encoded calcium indicators (GECIs) are protein-based Ca^2+^ sensors that couple Ca^2+^ binding to conformational changes in fluorescent protein modules, altering fluorescence intensity or FRET ratios to optically report Ca^2+^ dynamics (Lohr et al., 2021). GECIs are broadly classified into single-fluorescent and FRET-based dual-fluorescent types. Unlike exogenous small-molecule dyes, GECIs can be selectively expressed in specific cell types and further engineered to target subcellular compartments—such as soma, plasma membrane, endoplasmic reticulum (ER), or mitochondria—enabling precise monitoring of Ca^2+^ dynamics (Van Acker et al., 2019). Stobart et al. (2018b) demonstrated that membrane-anchored GECIs (e.g., Lck-GCaMP3/6) could capture fast, synaptically evoked Ca^2+^ transients in astrocytic processes and endfeet with high signal-to-noise ratios, clearly distinguishing these rapid events from the slower, soma-centered IP3R-mediated Ca^2+^ waves, highlighting the value of subcellular targeting in resolving Ca^2+^ signal heterogeneity. Using cytosolic AAV5-GfaABC1D-cyto-GCaMP6f-SV40, Srinivasan (Srinivasan et al., 2015) showed that Ca^2+^ dynamics differ between soma, primary processes, and microdomains of astrocytes; in IP3R2^–/–^ mice, somatic Ca^2+^ activity frequency was significantly reduced, whereas microdomain activity remained largely unchanged. Chai et al. (2017) further reported region-specific differences in spontaneous and evoked astrocytic Ca^2+^ activity between hippocampus and striatum. Haidey et al. (2021) compared endfoot Ca^2+^ dynamics reported by membrane-anchored GCaMP versus patch-loaded Fluo-4 and found that GCaMP captured brief, vessel-contraction–related transients, whereas Fluo-4 showed more sustained, plateau-like elevations, indicating complementary yet distinct temporal and spatial reporting by different indicators. Recent advancements in GCaMP performance have further enhanced GECI utility (Zhang et al., 2023). The jGCaMP8 family overcomes previous kinetic limitations, exhibiting fast and linear responses: jGCaMP8s provides maximal single-spike fluorescence change with moderate decay suitable for quantitative analysis; jGCaMP8f and jGCaMP8m offer faster decay and higher dynamic range, ideal for tracking rapid neuronal spikes and fine temporal dynamics. Moreover, jGCaMP8 effectively captures transient Ca^2+^ signals in small subcellular structures such as dendritic spines and axons, and soma-targeted variants improve signal-to-noise ratios in dense neural networks.

GECIs can be delivered using adeno-associated viruses (AAVs) under astrocyte-specific promoters (e.g., GFAP, GfaABC1D, ALDH1L1) to label astrocytes in specific brain regions, or via Cre-dependent transgenic approaches for whole-brain targeting (Gorzo and Gordon, 2022). Compared with conventional GFAP promoters, GfaABC1D offers higher specificity and lower background. Zhang et al. (2022) employed GfaABC1D-driven AAVs to image astrocytic mitochondrial Ca^2+^ dynamics at single-mitochondrion resolution, capturing spontaneous Ca^2+^ fluctuations and demonstrating the sensitivity and spatiotemporal resolution achievable with subcellular-targeted GECIs. Collectively, GECI-based cell-type–specific labeling and subcellular targeting have become essential tools for dissecting the complexity and functional diversity of astrocytic Ca^2+^ signaling.

#### 3.1.2 Imaging platforms

Investigating astrocyte morphology and Ca^2+^ dynamics relies on a variety of microscopy techniques, including confocal laser scanning microscopy (CLSM), two-photon laser scanning microscopy (2PLSM), and stimulated emission depletion (STED) microscopy, each offering distinct advantages in spatial resolution, imaging depth, and dynamic capture capabilities.

##### 3.1.2.1 Laser scanning confocal microscopy (LSCM)

Laser scanning confocal microscopy (LSCM) has become a widely used tool for studying astrocyte morphology and Ca^2+^ dynamics due to its accessibility, high planar resolution, and ease of use (Shigetomi et al., 2016). LSCM employs single-photon short-wavelength excitation and pinhole-based optical sectioning to reject out-of-focus fluorescence, producing high-resolution optical slices suitable for cultured cells and acute slices near the tissue surface (Cole et al., 2011; Pérez-Alvarez et al., 2013; Rosenegger et al., 2014). For instance, in Alzheimer’s disease hippocampal tissue, multichannel immunofluorescence combined with LSCM enables mapping the spatial distribution of inflammatory markers such as C1q and C3 near plaques versus control regions, and examining their association with astrocytes (Fixemer et al., 2025). Pirnat et al. (2021) applied Fura-2AM with alternating 340/380 nm excitation and dual-point calibration to calculate absolute intracellular Ca^2+^ concentration, including baseline, peak, and area under the curve (AUC). Concurrently, Fluo-4AM was imaged on a confocal microscope with 488 nm excitation using 20 × objectives for global observation and 63 × objectives for microdomain imaging at ∼1 Hz sampling to capture somatic and process-localized microdomain Ca^2+^ transients. Their results revealed significant differences in microdomain Ca^2+^ dynamics under varying culture conditions: astrocytes in neuron-conditioned medium (NB+) exhibited elevated resting Ca^2+^, increased microdomain peak frequency, and predominantly sustained ATP responses, whereas cells in Dulbecco’s modified Eagle medium (DMEM+) were prone to short oscillatory events. These findings highlight the utility of LSCM in linking ionic dynamics to cellular functional mechanisms.

##### 3.1.2.2 Two-photon laser scanning microscopy (2PLSM)

Two-photon laser scanning microscopy (2PLSM) utilizes near-infrared excitation to achieve nonlinear two-photon absorption, offering superior tissue penetration and reduced phototoxicity, making it well-suited for deep imaging *in vivo* or in thick tissue slices (Gorzo and Gordon, 2022). Compared to LSCM, 2PLSM can acquire high signal-to-noise ratio images at depths of several hundred micrometers and is widely employed to record astrocyte Ca^2+^ dynamics and morphological reconstructions in intact tissue (Svoboda and Yasuda, 2006). For example, in the mouse somatosensory cortex, 2PLSM combined with subcellularly targeted genetically encoded Ca^2+^ indicators—cytosolic GCaMP6f and ER-targeted G-CEPIA1er—allows precise focal-plane excitation, minimizing photodamage while penetrating deeply into tissue. Offline analysis of fluorescence signals from the soma, proximal processes, and microdomains enables dissection of astrocyte Ca^2+^ dynamics across compartments, revealing their roles in synaptic modulation and ER Ca^2+^ homeostasis (Lia and Zonta, 2025). Sun et al. (2022) used 2PLSM to perform 3D Ca^2+^ imaging of astrocytes in awake and anesthetized mice, finding that Ca^2+^ signals in the awake state were concentrated in somata and proximal regions, whereas anesthesia reduced overall Ca^2+^ activity and produced hotspots in distal somatic regions. These observations indicate that astrocyte Ca^2+^ signaling is not only modulated by physiological state but also exhibits pronounced spatial remodeling. Compared with conventional 2D scanning, 2PLSM enables visualization of functionally distinct hotspots along the z-axis, with quantitative characterization of spatiotemporal features using metrics such as distribution indices and centroid trajectories (Bindocci et al., 2017).

##### 3.1.2.3 Stimulated emission depletion (STED) microscopy

Stimulated emission depletion (STED) microscopy offers superior spatial resolution, high imaging speed, imaging depth, and signal-to-noise ratio, enabling visualization of fine astrocytic processes beyond the optical diffraction limit in live tissue (Tønnesen and Nägerl, 2013; Arizono and Nägerl, 2022). Astrocyte processes, particularly perisynaptic astrocytic processes (PAPs), are nanometer-scale structures; STED allows clear visualization of their ring-like morphology, nodes, and spatial relationship with synapses. It should be clarified that STED microscopy has not been used to directly monitor astrocytic Ca^2+^ signals. Instead, as shown by Arizono et al. (2020), STED has been combined with two-photon Ca^2+^ imaging to correlate the ultrastructure of perisynaptic astrocytic processes (PAPs) with Ca^2+^ dynamics. Henneberger et al. (2020) combined STED with dual-color labeling and patch-clamp recordings to study PAPs, revealing that long-term potentiation (LTP) induced retraction of PAPs near dendritic spines, accompanied by localized Ca^2+^ signal changes at the retraction nodes, correlated with spine enlargement. This demonstrates that STED can capture the structural plasticity of PAPs and the tight coupling of Ca^2+^ dynamics with synaptic function. However, STED imaging requires prolonged illumination, which can induce fluorophore photobleaching and phototoxicity, limiting its application for rapid dynamic processes.

In summary, these three imaging modalities offer complementary strengths. LSCM, relying on single-photon excitation and pinhole rejection of out-of-focus light, is suitable for cultured cells or thin slices, providing high-resolution structural imaging and microdomain Ca^2+^ signals, but it is limited by a small field of view, relatively slow imaging speed, and potential phototoxicity or photobleaching during long-term illumination (Pham et al., 2020). 2PLSM employs near-infrared two-photon absorption, allowing deep imaging in thick slices or *in vivo* while maintaining low phototoxicity, making it ideal for dynamic monitoring of subcellular or *in vivo* Ca^2+^ distribution (Theer et al., 2003; Lia and Zonta, 2025). STED overcomes the diffraction limit, achieving nanoscale resolution that clearly visualizes the ring-like morphology and nodes of PAPs and their spatial relationship with synapses; however, its complex operation, high equipment cost, and the loss of fluorescence signal along with phototoxicity still limit the observation of fast dynamic events (Chéreau et al., 2015).

### 3.2 Ca^2+^ Signal analysis

#### 3.2.1 ROI-based methods (regions of interest, ROIs)

Currently, analysis of astrocytic Ca^2+^ signals largely relies on region-of-interest (ROI)-based strategies. Representative software includes GECIquant (Srinivasan et al., 2015), which detects Ca^2+^ transients within somata, primary processes, and microdomains using thresholding and outputs time-series fluorescence data. CaSCaDe (Agarwal et al., 2017) incorporates machine learning algorithms to automatically denoise, binarize, and generate spatial event maps, enabling quantification of event frequency, amplitude, and duration. CHIPS (Barrett et al., 2018), an open-source toolbox, integrates multiple algorithms while providing motion correction and denoising functions, streamlining the analysis workflow. These ROI-based approaches have advanced quantitative Ca^2+^ signal analysis; however, they remain limited by the use of fixed ROIs (Bindocci et al., 2017). Microdomain Ca^2+^ events may extend beyond predefined boundaries, resulting in signal splitting or contamination of adjacent regions, while larger ROIs can underestimate local signal amplitudes. Thus, although ROI-based methods have played a pivotal role in early astrocytic Ca^2+^ research, their spatial flexibility is limited, highlighting the need for more dynamic, event-driven analytical strategies.

#### 3.2.2 Event-based methods (astrocyte quantitative analysis, AQuA)

To overcome the limitations of ROI-based approaches, AQuA introduces an event-driven analytical paradigm that directly detects and tracks Ca^2+^ events in spatiotemporal space. This approach identifies complex signal “fingerprints” that may propagate, contract, or deform, reconstructing their spatial extent and temporal boundaries (Wang et al., 2019). In its original implementation, AQuA can process both *in vitro* and *in vivo* datasets, allowing parameter presets based on indicator type and signal-to-noise ratio. Event detection is performed using thresholding, smoothing, and dedicated tracking algorithms, outputting rich metrics including area, amplitude, duration, frequency, and propagation characteristics. This not only overcomes the static limitations of ROIs but also enables single-cell resolution analyses of physiological heterogeneity, anatomical directionality of *in vivo* Ca^2+^ bursts, and direct correlation of Ca^2+^ events with extracellular neurotransmitter dynamics, providing a robust, unbiased quantitative tool for dissecting the spatiotemporal roles of astrocytes in neural circuit function. Recent developments with AQuA2 (Mi et al., 2025) have further enhanced its practical utility: across three independent datasets—*ex vivo* recordings, synthetic data with ground truth, and *in vivo* imaging (spinal cord/zebrafish)—AQuA2 substantially reduced false positives and over-segmentation, while doubling detection speed under typical conditions (and achieving up to several-fold improvements in complex patterns). This performance gain is attributed to the newly introduced propagation-alignment algorithm (BILCO) and a more robust higher-level framework, making AQuA2 superior to conventional ROI-based tools and other event-based methods in handling variable footprints and propagative signals, with fewer mergers and artifacts.

## 4 Functional roles of astrocytic Ca^2+^ signaling

Astrocytic Ca^2+^ signaling exerts multi-level regulatory functions, primarily including modulation of synaptic activity, regulation of neuroinflammation, dynamic control of energy metabolism, and neurovascular coupling.

### 4.1 Modulation of synaptic function

Astrocytic Ca^2+^ signals are intimately linked to synaptic function. At excitatory synapses, elevated astrocytic Ca^2+^ can activate Connexin 43 (Cx43) hemichannels, enhancing NMDA receptor-mediated excitatory postsynaptic currents (EPSCs) in pyramidal neurons, a process associated with astrocytic release of D-serine (Meunier et al., 2017). At inhibitory synapses, increases in astrocytic Ca^2+^ trigger calcium-dependent ATP release, which is extracellularly converted to adenosine to selectively activate adenosine A2A receptors, thereby inducing inhibitory postsynaptic currents (IPSCs) (Noriega-Prieto et al., 2021). Astrocytic Ca^2+^ signals regulate basal neurotransmission by modulating presynaptic release probability rather than the amplitude of individual events, thereby influencing synaptic plasticity. In cerebellar granule layer slices, local Ca^2+^ transients in astrocytic processes depend on P2Y1 receptors and IP3R2-mediated endoplasmic reticulum Ca^2+^ release, and blocking these signals reduces the release probability of nearby synapses without affecting postsynaptic amplitudes (Di Castro et al., 2011). Similarly, chelation of astrocytic Ca^2+^ in hippocampal CA1 increases mEPSC failure rates while leaving amplitudes unchanged, supporting tonic presynaptic regulation (Panatier et al., 2011). NMDA receptor- and L-VGCC-dependent membrane depolarization and Ca^2+^ influx in astrocytes decorrelate presynaptic release probability across convergent inputs, maintaining heterogeneity of baseline synaptic strength (Letellier et al., 2016). Moreover, NMDA receptor-induced presynaptic inhibition, reflected as decreased mEPSC frequency without amplitude changes, requires astrocytic Ca^2+^; blocking astrocytic Ca^2+^, metabolism, or L-VGCCs reverses inhibition and can increase release frequency, indicating that astrocytic Ca^2+^ is essential for NMDA-dependent presynaptic plasticity (Letellier and Goda, 2023). Aberrant astrocytic Ca^2+^ activity impairs LTD and can lead to cognitive deficits (Kang et al., 2025), emphasizing its critical role in synaptic plasticity and neural information processing.

### 4.2 Regulation of neuroinflammation

Neuroinflammation is an immune response in the central nervous system driven by glial cell activation, and astrocytic Ca^2+^ signaling plays a pivotal role in its regulation (Fairless et al., 2014). Experimental evidence indicates that G protein–coupled receptor (GPCR)-mediated intracellular Ca^2+^ elevations trigger two distinct types of vesicular release: (1) VNUT (vesicular nucleotide transporter, encoded by SLC17A9)-dependent ATP/ADP release, which activates microglia via P2X/P2Y receptors and induces pro-inflammatory cytokine production; and (2) VNUT-independent glutamate release, which acts on NMDA and mGluR receptors to induce neuronal hyperexcitability and oxidative stress, thereby indirectly amplifying neuroinflammation (Li H. et al., 2025). Sanjay et al. (2024) further demonstrated that IP3R2-mediated astrocytic Ca^2+^ signaling is a key driver of both ATP and glutamate vesicular release, amplifying neuroinflammatory responses via P2 and NMDA/mGluR signaling pathways. Pathological stimuli can deplete ER Ca^2+^ via GPCR activation, triggering STIM-mediated gating of membrane Orai1 channels and thereby inducing store-operated calcium entry (SOCE), which sustains and enhances microdomain Ca^2+^ oscillations in fine astrocytic processes (Novakovic and Prakriya, 2025). This Orai1-dependent Ca^2+^ influx not only amplifies Ca^2+^-dependent vesicular release (both VNUT-dependent ATP/ADP and VNUT-independent glutamate) but also modulates cellular metabolism and activates NF-κB and NLRP3 inflammasome pathways, effectively coupling intracellular Ca^2+^ dynamics to astrocytic inflammatory output (Novakovic and Prakriya, 2025). *In vivo* studies further support this notion: astrocyte-specific deletion of Orai1 suppresses inflammatory gene expression, reduces hippocampal inflammation induced by lipopolysaccharide (LPS), and alleviates depression-like behaviors, highlighting Orai1 as a central hub controlling astrocytic reactivity and neuroinflammation (Novakovic et al., 2023).

### 4.3 Regulation of energy metabolism

Astrocytes serve as the primary glycogen reservoir in the central nervous system, and their activity-dependent intracellular Ca^2+^ signaling rapidly regulates glycogenolysis and downstream energy metabolism. During periods of high energy demand, astrocytes utilize glycolysis to generate and release lactate, thereby supporting the metabolic needs of neighboring neurons (Pellerin and Magistretti, 1994; Pellerin and Magistretti, 2012). Norepinephrine acting through α1-adrenergic receptors (α1-ARs) coupled to Gq signaling induces intracellular Ca^2+^ elevation, while extracellular ATP can similarly trigger Ca^2+^ responses via P2X/P2Y receptors. Cross-talk between Ca^2+^ and cyclic adenosine monophosphate (cAMP) signaling further regulates the activities of glycogen phosphorylase and key glycolytic enzymes, enhancing intracellular glucose utilization and lactate production. Lactate is subsequently exported through monocarboxylate transporters (MCTs) for neuronal uptake (Hertz et al., 2010; Abbracchio et al., 2009; Coco et al., 2003; Pangršič et al., 2007). Horvat (Horvat et al., 2021) employed Fluo-4 Ca^2+^ indicators, FRET-based glucose probe FLII, and the lactate sensor Laconic to dissect these mechanisms. Their results revealed that β-adrenergic receptor (β-AR) activation alone, although elevating cAMP, was insufficient to significantly increase glucose uptake or lactate production. In contrast, α1-AR–dependent Ca^2+^ elevations more effectively raised intracellular glucose levels and induced glycolysis and lactate accumulation. Astrocytic Ca^2+^ signaling also promotes mitochondrial Ca^2+^ uptake, activating the tricarboxylic acid (TCA) cycle and oxidative phosphorylation to rapidly increase local ATP generation and meet the high energy demands of active synapses (Cabral-Costa and Kowaltowski, 2023). Notably, astrocytes from different brain regions and subtypes exhibit distinct Ca^2+^ buffering capacities—for example, cortical astrocytes display stronger Ca^2+^ buffering than striatal astrocytes (Huntington and Srinivasan, 2021)—which may underlie regional metabolic resilience and susceptibility to pathology. AMP-activated protein kinase (AMPK) acts as a key regulator of cellular energy metabolism and mitochondrial dynamics, playing a critical role in maintaining overall energy homeostasis. *In vitro* studies indicate that impaired lysosomal Ca^2+^ release leads to reduced CaMKK2 (Ca^2+^/calmodulin-dependent protein kinase kinase 2) expression and downstream p-AMPK inhibition, accompanied by decreased PFKFB3/NDUFS1 expression, impaired complex I activity, mitochondrial dysfunction (increased ROS and decreased membrane potential), and ATP depletion (Zhang et al., 2025). These findings underscore the central role of Ca^2+^ signaling in maintaining astrocytic energy homeostasis.

### 4.4 Regulation of neurovascular coupling

Neurovascular coupling (NVC) refers to the process by which neuronal activity regulates cerebral blood flow through astrocytes and local signaling molecules, thereby maintaining metabolic homeostasis in the brain (Tran, 2022). Evidence indicates that neuronal activation, via glutamate receptors such as mGluRs and AMPARs, elevates intracellular Ca^2+^ in astrocytes, which in turn triggers the release of vasoactive molecules including prostaglandin E2 (PGE2), epoxyeicosatrienoic acids (EETs), ATP, and adenosine, all of which play key roles in modulating local blood flow (Mishra et al., 2024). *In vivo* imaging studies using OGB-1-AM, Rhod-2-AM, or Fluo-4-AM to record astrocytic Ca^2+^ signals alongside vascular responses have reported inconsistent observations. Some studies detected rapid Ca^2+^ transients in astrocytic somata following sensory stimulation (Lind et al., 2013), whereas others reported sparse somatic Ca^2+^ elevations that lagged behind vascular changes (Tran et al., 2018; Nizar et al., 2013). These discrepancies may arise from several factors, including indicator sensitivity and subcellular targeting, temporal resolution and sampling volume of imaging, awake versus anesthetized states, differences in brain regions or stimulation paradigms, and threshold settings for detecting microdomain events during data analysis. Recent studies employing genetically encoded calcium indicators (GECIs) in adult mouse astrocyte processes revealed that somatic Ca^2+^ signals are high-threshold and infrequent, whereas microdomain Ca^2+^ signals in astrocytic processes are short-latency, reliable, and highly correlated with local blood flow changes (Otsu et al., 2015; Institoris et al., 2022). These findings suggest that astrocytes integrate local neuronal activity through microdomain Ca^2+^ signaling, which acts as a precise and rapid mediator of functional hyperemia. The spatiotemporal precision of these microdomain events enables faster integration of neuronal activity and initiation of vascular responses, highlighting microdomain Ca^2+^ as a critical signal for understanding the mechanisms of NVC.

## 5 Summary and perspectives

In summary, this review systematically highlights recent advances in astrocytic Ca^2+^ signaling, encompassing its sources, classifications, detection methodologies, and functional significance. Astrocytes finely tune Ca^2+^ transients through intracellular stores, such as IP3R2- or RyR-mediated endoplasmic reticulum release, and extracellular influx via TRP channels, NMDA receptors, or NCX, allowing dynamic responses to environmental cues and synaptic activity. Subcellular compartments display distinct Ca^2+^ dynamics: microdomains exhibit rapid, localized, second-scale signals, whereas somata show low-frequency, long-lasting whole-cell events. These compartmentalized dynamics provide astrocytes with the capacity for precise regulation of synaptic function, energy metabolism, neuroinflammation, and neurovascular coupling. Methodologically, the integration of high spatiotemporal resolution multimodal imaging with event-driven analytical frameworks offers a powerful approach for moving from correlative observation to causal mechanistic insight.

Despite these advances, several challenges remain. First, many studies still describe astrocytic Ca^2+^ dynamics at the “average cell” level, overlooking clear heterogeneity across brain regions, developmental stages, and pathological states, including differences in receptor/channel repertoires, organelle composition, and coupling modalities. Future work should aim to characterize genotype (single-cell or spatial transcriptomics), morphology (high-resolution structural imaging), and real-time function (organotypic Ca^2+^ dynamics) at the single-cell or subcellular scale (Batiuk et al., 2020; Aryal et al., 2022), enabling identification of astrocyte subpopulations such as “ER-dominant,” “mitochondria-coupled,” or “lysosome-triggered” types and assessment of their plasticity under physiological and pathological conditions. Second, astrocytic Ca^2+^ signals may encode information through frequency, phase, and spatial patterns to regulate synaptic and network activity while coordinating blood flow and metabolism (Bazargani and Attwell, 2016). However, the precise coding rules and functional readouts of these patterns remain largely unknown. Current imaging combined with electrophysiology still faces limitations in spatiotemporal resolution and causal verification. In disease contexts, aberrant Ca^2+^ signaling has been observed, including intercellular Ca^2+^ waves in epilepsy, temporal and amplitude imbalances in Alzheimer’s disease, and impaired metabolic clearance in sleep disorders Carmignoto and Haydon, 2012; Kuchibhotla et al., 2009; Hastings et al., 2023). Future studies should develop cross-modal, multi-scale synchronous measurements and causal manipulations, establish quantitative metrics, and perform cross-model comparisons to elucidate shared and disease-specific mechanisms of Ca^2+^ signaling. Third, astrocytic Ca^2+^ signaling serves as a critical hub linking neuronal network activity, blood flow, and metabolism, yet intervention studies remain limited. Certain bioactive compounds from traditional Chinese medicine may restore Ca^2+^ homeostasis via receptor, channel, and mitochondrial pathways, and acupuncture has been reported to remodel astrocyte–neuron Ca^2+^ dynamics and influence neurovascular coupling. Future research should leverage cross-modal synchronous detection and causal validation to elucidate how interventions, such as herbal compounds or acupuncture, modulate disease-associated Ca^2+^ dysregulation and explore their potential as individualized therapeutic biomarkers.
