# Correction: Advances in astrocytic calcium signaling research

**DOI:** 10.3389/fncel.2025.1764027

**Published:** 2026-01-05

**Authors:** Yuzhu Chen, Yejun Ye, Joyce Jia, Binhao Long, Tingting Dou, Xingke Yan

**Affiliations:** 1 Department of Traditional Chinese Medicine, Qinghai University School of Medicine, Xining, Qinghai, China; 2 College of Acupuncture and Tuina, Gansu University of Chinese Medicine, Lanzhou, Gansu, China

**Keywords:** astrocyte calcium signaling, review, astrocyte, calcium, calcium signaling

In the published article, the Funding source “the Gansu Provincial Higher Education Scientific Research Program (grant no. 2018-18C)” was erroneously omitted from the Funding statement.

The corrected **Funding** statement appears below.

The author(s) declared that financial support was received for this work and/or its publication. This work was supported by the 2023 Gansu Province Joint Scientific Research Fund, General Program (grant no. 23JRRA1522), the Gansu Provincial Higher Education Scientific Research Program (grant no. 2018-18C), the 2024 Traditional Chinese Medicine First-level Discipline “Qihuang Talent” Mentor Special Fund (grant no. ZYXKBD-202409), the 2023 Gansu University of Chinese Medicine Science and Innovation Fund (Grant No. 2023KCYB-2), the 2025 Gansu University of Chinese Medicine Graduate “Innovation and Entrepreneurship” Fund (grant no. 2025CXCY-002), and the 2025 Gansu Province University Teachers' Innovation Fund (grant no. 2025A-115).

In the published article, the affiliation “1. Department of Traditional Chinese Medicine, Qinghai University School of Medicine, Xining, Qinghai, China” was erroneously assigned to the Xingke Yan in the author list along with affiliation 2. Xingke Yan's affiliation has been corrected to:

Xingke Yan^2*^

^2^ College of Acupuncture and Tuina, Gansu University of Chinese Medicine, Lanzhou, Gansu, China

The original version of this article has been updated.

