# Correction: Advances in astrocytic calcium signaling research

**DOI:** 10.3389/fncel.2026.1821571

**Published:** 2026-03-16

**Authors:** Yuzhu Chen, Yejun Ye, Joyce Jia, Binhao Long, Tingting Dou, Xingke Yan

**Affiliations:** 1 Department of Traditional Chinese Medicine, Qinghai University School of Medicine, Xining, Qinghai, China; 2 College of Acupuncture and Tuina, Gansu University of Chinese Medicine, Lanzhou, Gansu, China

**Keywords:** astrocyte calcium signaling, review, astrocyte, calcium, calcium signaling

In the published article, there was a mistake in the **Funding** statement. The funding statement for the Gansu Provincial Higher Education Scientific Research Program was displayed as “2018-18C”. The correct statement is “the Gansu Provincial Higher Education Scientific Research Program (Grant No. 2018C-18).”

The original version of this article has been updated.

